# Tailoring the separation properties of flexible metal-organic frameworks using mechanical pressure

**DOI:** 10.1038/s41467-020-15036-y

**Published:** 2020-03-05

**Authors:** Nicolas Chanut, Aziz Ghoufi, Marie-Vanessa Coulet, Sandrine Bourrelly, Bodgan Kuchta, Guillaume Maurin, Philip L. Llewellyn

**Affiliations:** 1 Aix-Marseille University, CNRS Laboratoire MADIREL (UMR7246), Marseille, France; 2 Massachusetts Institute of Technology, MultiScale Materials Science for Energy & Environment <MSE 2>, MIT-CNRS-AMU joint laboratory/MIT Energy Initiative, Cambridge, 02139 MA USA; 3 Institut de Physique de Rennes, IPR UMR 6251 CNRS, Rennes, France; 4 Department of Chemistry, Wroclaw University of Science and Technology, Wroclaw, Poland; 5 Institut Charles Gerhardt Montpellier, UMR 5253 CNRS, UM, ENSCM, University of Montpellier, Montpellier, France

**Keywords:** Carbon capture and storage, Metal-organic frameworks

## Abstract

Metal-organic frameworks are widely considered for the separation of chemical mixtures due to their adjustable physical and chemical properties. However, while much effort is currently devoted to developing new adsorbents for a given separation, an ideal scenario would involve a single adsorbent for multiple separations. Porous materials exhibiting framework flexibility offer unique opportunities to tune these properties since the pore size and shape can be controlled by the application of external stimuli. Here, we establish a proof-of-concept for the molecular sieving separation of species with similar sizes (CO_2_/N_2_ and CO_2_/CH_4_), via precise mechanical control of the pore size aperture in a flexible metal-organic framework. Besides its infinite selectivity for the considered gas mixtures, this material shows excellent regeneration capability when releasing the external mechanical constraint. This strategy, combining an external stimulus applied to a structurally compliant adsorbent, offers a promising avenue for addressing some of the most challenging gas separations.

## Abstract (web summary)

Separation of gasses with similar physical and chemical properties can be energy demanding, and adsorption-based technologies may provide alternatives with lower energy consumption. Here, the authors show mechanical control of pore size aperture in flexible metal-organic frameworks for separation of gasses.

## Introduction

With an estimation that 10–15% of the world’s energy resources are used for separations of chemical mixtures^1^, it is clear that any improvement of these processes can have a significant impact from an economic and environmental standpoint. Gas separation and purification steps are particularly energy demanding, especially when the species to be separated exhibit similar physical and chemical/electronic properties. With a potential reduction in energy consumption estimated at 80%, adsorption-based technologies using porous solids or membranes are considered as an attractive alternative to costly distillation and absorption processes^2^.

The viability of adsorption-based separation processes relies on the development of adsorbents with specific physical and chemical properties, controlling the interactions with the molecules in the mixture to be separated. Depending on the process envisaged, adsorption-based separations can be ruled by various mechanisms: (i) enthalpic, where the separation is driven by the difference in affinities of the components in a mixture towards a given adsorbent (ii) kinetic, where the separation depends on the difference in diffusion rates of the various species through the porous material (iii) entropic, where the shape of the pores of the adsorbent proffers an optimal packing of a given molecule and (iv) molecular sieving, where the adsorbent has pores of specific dimensions that allow small molecules to enter whilst excluding larger ones^3^. While for each separation mechanism, specific physical and chemical properties of the adsorbent are required, there is a common quest for an optimal balance between the highest selectivity towards a given species combined with the lowest possible energy required for regeneration, i.e., desorption of confined species from the pores for further re-utilization of the adsorbent. However, the selectivity and regeneration performances are often antagonistic, and this makes the realization of such an optimal scenario quite complicated. From the aforementioned separation mechanisms, molecular sieving can be considered as the ultimate goal, since it allows the complete recovery of one component from a mixture leading to possible ideal selectivity. However, this type of separation is highly challenging to achieve for molecules of similar sizes^3^. One strategy that is currently adopted involves the development of ultra-microporous adsorbents with precisely tailored pore dimensions. Such a family of materials has successfully led to molecular sieving for complex separations of molecules with similar sizes, such as carbon dioxide/nitrogen^4^, propane/propylene^5^, and butane/isobutane^6^. However, each of these separations is material dependent and requires specific pore dimensions with the design of a novel porous material for each mixture of interest. Furthermore, the high degree of confinement in ultra-microporous adsorbents leads to strong adsorbate/adsorbent interactions. This has two consequences: (i) a significant amount of energy is required to induce gas desorption and (ii) a slow diffusion of species may limit their evacuation, both these effects leading to a costly regeneration of the adsorbent.

Here, we present a strategy where the pore size aperture of a flexible metal-organic framework (MOF) can be finely tuned via the application of external mechanical pressure. Specifically, the possibility to control in situ the guest-assisted breathing behavior of the MIL-53 framework during both the adsorption and desorption steps is presented.

**Fig. 1:**
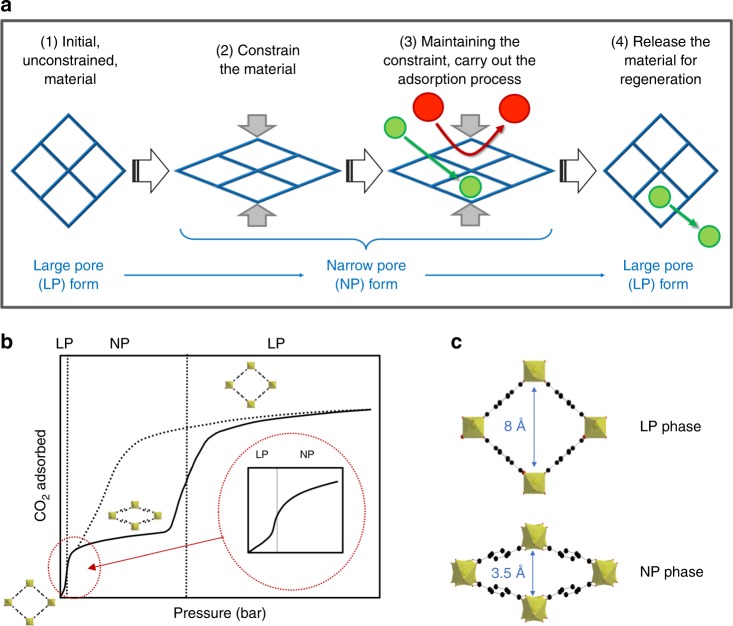
Flexibility of the MIL-53 framework. **a** Schematic representation of the concept developed in the present study: control of the breathing behavior of the flexible MIL-53 MOF by application of an external mechanical pressure to provoke a molecular-sieving type separation, followed by regeneration from the unconstrained MOF after release of the mechanical constraint. **b** CO_2_ isotherm (full-line—adsorption, dashes—desorption) of the unconstrained MIL-53 evidencing a two-step process indicative of the framework breathing. Phase transitions between large pore (LP) and narrow pore (NP) phases are indicated by vertical dashed black lines. **c** Evolution of pore size aperture between the LP and NP phases of MIL-53^21^.

Guest-assisted breathing phenomena in MOFs are well documented^7–10^. These entail through adsorption of a guest molecule, a concerted crystal-to-crystal phase transition between equilibrium structures, e.g., open and contracted phases, generally involving a significant change in the unit cell volume. Initially considered as a fundamental curiosity, this intriguing behavior has attracted much attention lately for both the storage and the separation of molecular species^11^. Further works have shown that such a magnitude of flexibility can equally be induced by other physical stimuli, such as temperature^12^, light^13^, electrical field^14^, and mechanical pressure^15^. In the latter case, some studies have highlighted the possibility of using the mechanically induced flexibility of MOFs to store energy mechanically^16–19^ or manage thermal effects in gas storage applications^20^. Nonetheless, to date, no study has explored this concept in a synergistic combination with adsorption for further development in separation-based processes. The concept proposed in this study is to (i) use mechanical pressure to induce the structural transition from an open-pore form of a compliant MOF towards a contracted form, then (ii) proceed to adsorption with optimal conditions of separation, before (iii) switching back to the open pore form by releasing the mechanical pressure for regeneration in a less confined state (Fig. 1a).

## Results

### Experimental approach

The porous MOF MIL-53^21^ has been selected as a model candidate to propose a proof-of-concept of this line of thought. This channel-like framework made of chains of metal octahedra sharing μ_2_-OH vertices linked through terephthalate ligands, has been demonstrated to exhibit in its aluminum and chromium forms a guest-assisted reversible phase transition between a large pore (LP) and a narrow pore (NP) phase, accompanied with a remarkable change of the unit cell volume of about 40%^22^. As illustrated in Fig. 1b for CO_2_ adsorption, this leads to a two-step isotherm. At room temperature and in the absence of guest molecules, the LP phase is the most stable form. A slight increase in pressure induces a narrowing of the pores leading to the NP phase while increasing the CO_2_ pressure reopens the structure in its LP phase. On desorption, as the CO_2_ is evacuated from the pores, the reversible structural transition is observed (i.e., LP to NP phase) with hysteresis in the transition pressure.

As a consequence of this phase transition, the pore size aperture varies from 8 Å in the LP phase down to 3.5 Å in the NP phase (Fig. 1c), making this material potentially of interest to molecular sieve CO_2_ over N_2_ and CH_4_ due to the difference in their kinetic diameters (3.3, 3.7, and 3.8 Å, respectively)^23^. Furthermore, previous studies have demonstrated that a similar contraction of the structure can be induced by the application of hydrostatic mechanical pressure in the range of 20–100 MPa^15,17^. These structural features, together with the excellent mechanical stability of MIL-53(Al, Cr) up to 500 MPa^18^, make this MOF ideal for the present study. To exploit this concept, we have developed a unique experimental setup allowing the measurement of high-pressure gas adsorption isotherms under external, uniaxial mechanical constraint (Supplementary Fig. 1). The use of a hydraulic mechanical press allows for the control of the mechanical pressure, which is maintained constant and continuously applied on the powder bed throughout the adsorption experiment.

### Gas adsorption under applied mechanical pressure

**Fig. 2:**
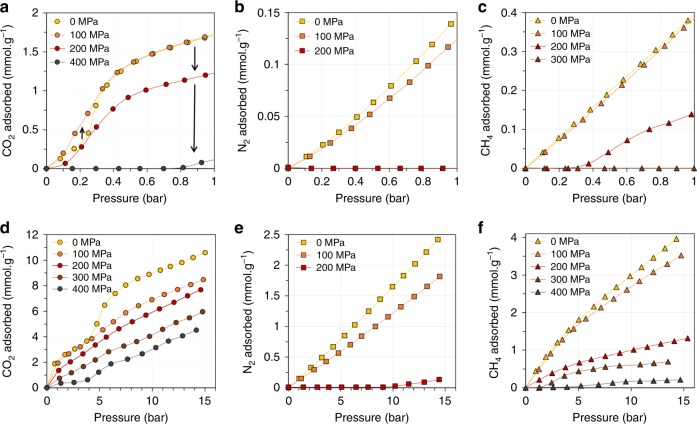
Effect of mechanical pressure on CO_2_, N_2_, and CH_4_ adsorption properties of MIL-53(Al). **a**–**f** Adsorption isotherms of CO_2_ (**a**, **d** circles), N_2_ (**b**, **e** squares), and CH_4_ (**c**, **f** triangles) recorded at 303 K for different applied mechanical pressures. **a**–**c** correspond to isotherms recorded up to 1 bar while **d**–**f** correspond to isotherms recorded up to 15 bar. Yellow, orange, red, brown, and dark gray filled symbols represent an applied mechanical pressure of 0, 100, 200, 300, and 400 MPa, respectively. **a** Black arrows indicate the evolution of CO_2_ uptake when gradually increasing the mechanical constraint.

Figure 2 reports the adsorption isotherms of CO_2_, N_2_, and CH_4_ collected on the MIL-53(Al) constrained to various mechanical pressures at 303 K. The unconstrained MIL-53(Al) presents the above-mentioned two-step isotherm for CO_2_ in agreement with previous studies^22^. Unlike carbon dioxide, nitrogen and methane do not trigger the breathing transition in the temperature and gas pressure conditions investigated here (*T* = 303 K and *P*_gas_ ≤ 15 bar) and the material remains in its LP phase as previously evidenced by X-ray diffraction (XRD)^24^.

Interestingly, the application of a mechanical pressure of 100 MPa shows an enhancement of the CO_2_ uptake by a factor two at *P*_CO2_ around 0.2 bar (Fig. 2a). This suggests a transition from the LP to the NP phase, since previous microcalorimetry and molecular modeling studies have shown that the interaction energy of CO_2_ in the NP phase is significantly higher than in the LP phase, leading to higher uptake at low pressure^22,25–28^. However, it is well documented that for a granular medium under uniaxial constraint, the mechanical pressure is heterogeneously distributed on the different crystallites through the existence of force chains^29,30^. This suggests that not all the crystallites have undergone the LP to NP phase transition and therefore that both phases coexist, which is confirmed by the step in CO_2_ uptake above *P*_CO2_ = 4–5 bar (Fig. 2d), the pressure corresponding to the reopening of the structure (NP to LP phase transition).

**Fig. 3:**
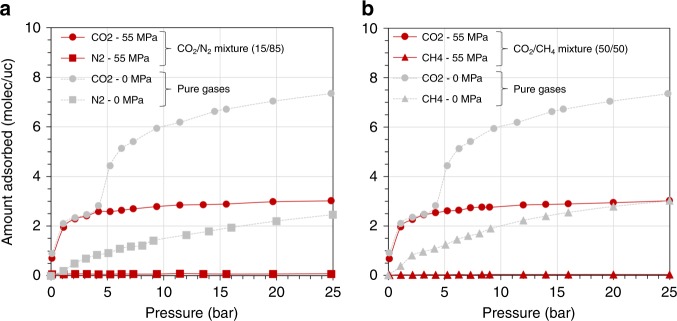
Hybrid osmotic Monte Carlo (HOMC) simulations of binary mixtures in MIL-53(Cr). **a**, **b** Simulated isotherms at 298 K for **a** the binary mixture CO_2_/N_2_ (15/85) under mechanical constraint (red circles—CO_2_; red squares—N_2_), and pure CO_2_ (gray circles) and N_2_ (gray squares) on the unconstrained MIL-53(Cr) and **b** the binary mixture CO_2_/CH_4_ (50/50) under mechanical constraint (red circles—CO_2_; red triangles—CH_4_), and pure CO_2_ (gray circles) and CH_4_ (gray triangles) on the unconstrained MIL-53(Cr). Isotherms were obtained by using hybrid osmotic Monte Carlo simulations with an applied isostatic mechanical pressure of 0 and 55 MPa for the unconstrained and mechanically constrained MIL-53(Cr), respectively. The unit of the *y*-axis ‘molec/uc’ refers to molecules per unit cell.

Interestingly, this step disappears when applying a mechanical pressure of 200 MPa. We relate this result to the fact that, under such large mechanical pressure, sintering of the powder occurs, i.e., we observe the irreversible merging of the powder into a pellet. This concerted closure of the crystallites is confirmed by the N_2_ isotherm collected under 200 MPa (Fig. 3b, e). Significantly, no uptake is observed until *P*_N2_ around 10 bar indicating that all the crystallites are maintained in a form, which does not allow N_2_ to enter the pores in this gas pressure range. However, beyond 10 bar, the slight increase in N_2_ uptake is most probably associated with a reopening of certain crystallites. This highlights a competition between the external mechanical pressure that tends to close the pores and the internal pressure due to gas adsorption that would tend to open them. It is thus clear that an equilibrium state exists in the bulk powder that results from a subtle balance between these two effects. It is important to note the excellent reproducibility of the adsorption isotherms after loading/unloading cycles (Supplementary Fig. 2), together with the excellent stability of the structure as verified by the XRD data collected before and after 300 cycles (Supplementary Fig. 3).

Similar behavior to nitrogen is observed for methane adsorption under mechanical pressure. Indeed, the uptake is negligible until (i) *P*_CH4_ around 0.2 bar for a mechanical pressure of 200 MPa, (ii) *P*_CH4_ around 2 bar for a mechanical pressure of 300 MPa and (iii) *P*_CH4_ around 5 bar for a mechanical pressure of 400 MPa (Fig. 2c, f). The higher mechanical pressure necessary to maintain the structure inaccessible to CH_4_ molecules suggests a slightly higher internal stress induced by CH_4_ compared to N_2_. As in the case of CO_2_ (Fig. 2d), increases in mechanical pressure to 300 and 400 MPa lead to a decrease in CH_4_ uptake indicating a gradual compression of the structure due to the mechanical pressure rather than the existence of a fully contracted pore structure.

### Separation properties from single component gas adsorption

From the isotherms obtained on the three gases, the separation factors for CO_2_/N_2_ (15/85) and CO_2_/CH_4_ (50/50) mixtures at 1 bar, 303 K, and under increasing mechanical constraint have been calculated. The separation factor is defined as the ratio of the amount adsorbed of one gas with respect to a second at their given partial pressures^31^, providing a valuable estimation of the separation properties of an adsorbent for a given mixture. For both separation considered, we can see that the application of mechanical pressure on the MIL-53(Al) leads to an increase of the separation factor (from 2.2 to 3 for CO_2_/N_2_ (15/85) under 0 and 100 MPa, respectively, and from 6.8 to 7.2 and 21.1 for CO_2_/CH_4_ (50/50) under 0, 100, and 200 MPa, respectively). More significantly, the corresponding separation factor values reach infinity above 200 and 300 MPa for CO_2_/N_2_ and CO_2_/CH_4_, respectively, since in these conditions the gas uptake is negligible for both N_2_ and CH_4_.

An interesting approach to induce molecular sieving at sub-atmospheric pressure using the MIL-53(Al) has previously been proposed by Mishra et al.^32^. In this work, the authors carried out CO_2_ adsorption, and on desorption the authors did not fully reactivate the sample to the LP form. Thus, with the sample in its NP form, single-component gas adsorption isotherms evidenced negligible uptake of several gases (N_2_, CH_4_, O_2_, and CO). However, the range of pressure where molecular sieving can be considered is limited (around 0.6 bar for N_2_ and CH_4_) compared to the mechanical approach proposed here (until 10 bar at 200 MPa for N_2_ and until 5 bar at 400 MPa for CH_4_). Also, as opposed to the work of Mishra et al. that used the non-reversible CO_2_ desorption behavior in the particular case of MIL-53(Al), our approach could be applied to any type of flexible adsorbents able to withstand enough mechanical pressure in order to tune their separation properties in the required gas pressure range.

The experimental results presented so far highlight the possibility to finely tune the pore size aperture of the MIL-53(Al) depending on the external mechanical pressure applied. This allowed us to control the accessibility of a given molecule to its microporosity, potentially leading to molecular sieving separations. However, caution must be taken when using single-component gas adsorption isotherms to predict the adsorption of gas mixtures in flexible MOFs as structural changes of the adsorbent may occur at different pressures for the two adsorbates^33^.

### Separation of CO_2_/N_2_ and CO_2_/CH_4_ binary mixtures

A hybrid osmotic Monte Carlo (HOMC) simulation strategy has therefore been deployed to mimic the experimental scenario, i.e., the mechanically constrained structure is freely relaxed at all stages of adsorption. These calculations were performed on the MIL-53 framework in its Cr-version for two reasons: (i) MIL-53(Al) and MIL-53(Cr) show both similar two-step CO_2_ adsorption isotherms and mechanical resilience under the application of an external pressure and (ii) our previously derived flexible force field for the MIL-53 framework was fully validated for the Cr-version^34^. Indeed, a NP to LP phase transition of MIL-53(Cr) was predicted upon CO_2_ adsorption at about 5 bar in excellent agreement with the corresponding experimental data^22^ or under the application of an external mechanical pressure of at least 55 MPa^34^. This magnitude of mechanical constraint was thus applied isostatically to the MIL-53(Cr) structure during the adsorption of a CO_2_/N_2_ mixture (molar composition 15/85) and a CO_2_/CH_4_ mixture (molar composition 50/50) at 298 K.

Figure 3 shows that for both mixtures, the amount of N_2_ and CH_4_ adsorbed by the mechanically constrained MIL-53(Cr) is negligible, while the amount of CO_2_ adsorbed is similar to the uptake corresponding to the filling of the NP phase. This result emphasizes an almost infinite CO_2_/N_2_ and CO_2_/CH_4_ selectivity when the sample is mechanically constrained, and this remains true even at high pressure up to 25 bar. In both cases, these simulations evidenced that the structure is maintained in a NP phase over the whole range of pressure with a unit cell volume of 1084 Å^3^ associated with a pore size of 3.5 Å, equivalent to the dimensions of the CO_2_-loaded NP phase (1072 Å^3^) previously reported experimentally^36^ and theoretically^34,35^. This confirms that the separation is driven by a size exclusion effect that hampers the adsorption of N_2_ and CH_4_ of larger kinetic diameters into the NP phase. This whole set of predictions reveals that the mechanically constrained MIL-53 is an excellent candidate for the separation of these two strategic mixtures. An adsorption cell for gas-mixture breakthrough experiments is currently under development to experimentally validate these findings.

### Regeneration of the adsorbent

**Fig. 4:**
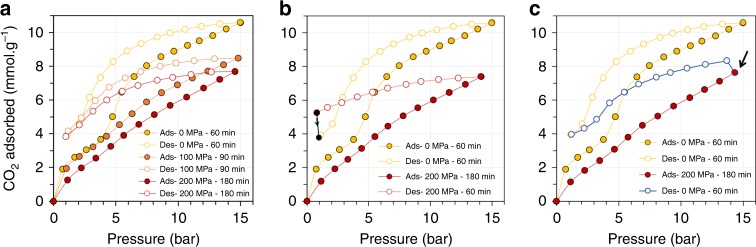
Regeneration of MIL-53(Al). **a**–**c** CO_2_ adsorption–desorption isotherms at 303 K **a** for the unconstrained MIL-53(Al) (yellow), and under a mechanical constraint of 100 (orange) and 200 MPa (red) with an equilibrium time set at 60, 90, and 180 minutes per point, respectively **b** under 200 MPa with an equilibrium time set at 180 minutes per point for adsorption (red full line) and 60 minutes per point for desorption (dashed red line). Filled black circles indicate the evolution of CO_2_ uptake when allowing the system to further equilibrate for 10 hours **c** under 200 MPa with an equilibrium set at 180 minutes per point for adsorption (red full line) and after release of the mechanical constraint under gas pressure with an equilibrium time for desorption set at 60 minutes per point (blue line). The black arrow indicates the point on the isotherm at which the mechanical pressure has been released. **b**, **c** CO_2_ adsorption–desorption isotherm for the unconstrained MIL-53(Al) (yellow) with an equilibrium time set at 60 minutes is given for comparison. **a**–**c** Filled and open circles represent adsorption and desorption, respectively.

The other major point of our approach concerns the possibility to regenerate the material after releasing the mechanical constraint, and the desorption branches of the isotherms are key to follow for that purpose. To study the regeneration capability of the MIL-53(Al), CO_2_ adsorption–desorption isotherms for the unconstrained material and under various mechanical constraints were thus measured (Fig. 4a).

The isotherms obtained on the MIL-53(Al) under various mechanical constraints all display hysteresis loops indicating the presence of energy barriers for desorption as previously described^22^. Comparing the results obtained with and without mechanical constraint, we observe that the amount of CO_2_ remaining in the pores after desorption to 1 bar is comparable (Fig. 4a). This indicates that CO_2_ can be desorbed from the material, whether under mechanical constraint or not. However, when increasing the mechanical pressure, the equilibrium time for adsorption/desorption becomes increasingly longer. Although it is beyond the scope of the current paper, this indicates that kinetic separations could also be envisaged with this technique as the mechanical pressure allows for a control of the mass transfer. With this in mind, the effect of releasing the mechanical pressure on the desorption diffusion rates was investigated.

Figure 4b shows the CO_2_ adsorption–desorption isotherm recorded on the MIL-53(Al) for the unconstrained material in which a desorption time of 1 hour per point was large enough to ensure equilibrium. Adsorption isotherms are also shown for the material under constant mechanical constraint of 200 MPa, where 3 hours are required to ensure full equilibrium. However, if the desorption time is set at 1 hour for the mechanically constrained material, it can be seen that the pore emptying is not complete at the final desorption point, confirming that equilibrium is not reached (thus the dashed line). Indeed, after this final point, around 10 hours were required for the sample to finally reach equilibrium, as represented by the black filled circles. Whilst 3 hours are required to ensure full equilibrium for the mechanically constrained MIL-53(Al) under 200 MPa, 80% of the gas is adsorbed in less than 30 minutes and 70% in less than 15 minutes (Supplementary Fig. 4), an important point looking at the application of such material in swing adsorption processes.

In a second experiment, the adsorption was again carried out under a mechanical constraint of 200 MPa, but the constraint was released at the end of the adsorption step (Fig. 4c). At this point, a slight increase in uptake is observed, which can be attributed to a relaxation of the structure to accommodate some more CO_2_. From this point onwards, we used a desorption time of 1 hour per point similar to the experiment with the unconstrained material, and it can be observed that the amount adsorbed after desorption exactly matches that for the unconstrained sample. This is a key result indicating that the regeneration properties of the adsorbent are retrieved when the mechanical pressure is released.

## Discussion

This fundamental exploration of the adsorption behavior of the flexible MIL-53 under mechanical constraint demonstrates the possibility to finely tune its pore size aperture during both the adsorption and desorption processes. The fine control of the pore size with the magnitude of applied mechanical pressure allows (i) the separation properties to be optimized for a given mixture, while (ii) improving the regeneration capability and the desorption diffusion rates of flexibles MOFs compared to rigid ultra-microporous materials. Such methodology is highly attractive for the molecular sieving separations of the most challenging gas mixtures, but further works have to be conducted to study its large-scale application. The key challenge to address is to ensure a uniform distribution of the mechanical pressure on the powder bed at a large scale, in order to spatially achieve similar separation properties in the adsorption column. Nonetheless, the proof-of-concept presented here highlights the possibility to precisely and reversibly reconfigure the microporous network of flexible MOFs in response to an external stimulus. This approach paves the way for the development of new separation processes, but could also be relevant for any applications where the in situ control of an absorbent porosity is of interest.

## Methods

### Adsorption measurements under applied mechanical pressure

An experimental setup for the in situ measurement of gas adsorption isotherms under controlled mechanical constraint was specially designed and used (Supplementary Fig. 1). First, a sample-cell has been developed in order to maintain the gas pressure when applying an external mechanical constraint (Supplementary Fig. 1—Photo). The cell is based on a die press used to make pellets for infrared spectroscopy with the particularity to use O-rings to maintain the system leak-tight to gas pressure. An inlet and valve system to allow vacuum and gas input has equally been built onto the cell. The leak tests have shown a perfect sealing until 20 bar. A CrushIR digital hydraulic press from PIKE technologies is used to load the mechanical pressure up to 15 ton. This is equivalent to around 3000 MPa on the sample as the contact surface of the piston is of 0.5 cm² (1 ton cm² = 98.1 MPa).

Prior to the introduction in the cell, the MIL-53(Al) sample prepared following the synthesis route previously reported^21^ is activated ex situ at 473 K for 16 h under a dynamic vacuum of 10^−3^ mbar. It is then transferred to the sample-cell in a glovebox to avoid any contamination from the ambient atmosphere. The cell is placed under the hydraulic press and then connected to an adsorption manometry system for the measurement of adsorption isotherms. The entire system is located in a thermally controlled cage, and the temperature maintained at 303 K. The gas dosing system was designed and constructed in-house using Swagelok VCR 1/4″ stainless steel components with a series of pneumatic valves and consists of a gas inlet line, a measurement manifold and a gas evacuation system. The leak-tightness is ensured by copper ring seals, and the system is designed to withstand pressures up to 200 bar, although the pressure gauges currently installed (Mensor CPT6000 series) have a maximum pressure of 40 bar. The gas injection system is completely automated via homemade software and interface.

The measurement manifold consists of the sample cell and a reference volume with both a low-pressure gauge (to 1 bar) and a high-pressure gauge (from 1 to 40 bar) for optimal accuracy on the entire pressure range. The reference volume was first determined by the expansion of He from a calibrated volume of 26,069 cm^3^. The adsorption measurements are carried out based on the standard manometric adsorption technique. A gas dose is introduced into the reference volume through valve V2 and allowed to stabilize before being brought into contact with the sample by opening valve V3 (Supplementary Fig. 1). The amount of gas introduced into the reference volume and remaining in the system after adsorption is calculated via REFPROP software, and the amount adsorbed is determined from the mass balance. In order to calculate the amount adsorbed, it is equally necessary to know the precise dead volume of the cell containing the sample. This is obtained by an experiment involving an inert gas, which is assumed not to adsorb (Helium) prior to each adsorption experiment.

### X-ray diffraction

XRD patterns were acquired using a powder INEL diffractometer at CINAM laboratory (UMR 7325 CNRS AMU). The incident beam was monochromatic with CuKα1 radiation (*λ* = 1.5406 Å). The samples were introduced into thin-walled glass capillaries (inner diameter 0.4 mm). The sample impregnation in ethanol was done in order to fully open the structure (LP phase) and avoid any LP/NP phase coexistence. It was done directly by dropping a few droplets of ultrapure ethanol inside the capillary. Data collection was done using a curved position sensitive detector over a total angular range 2*θ* between 0.29° and 107.42° by steps of 0.029°. During the acquisition, the capillary was mounted on a rotating holder to avoid any preferential orientation. The acquisition time was set to 1800 s.

### Modeling

The simulation box consisted of 32 unit cells of the conventional LP structure of MIL-53(Cr) built from the crystallographic coordinates previously reported by X-ray powder diffraction (XRPD) study^21^. The corresponding box lengths are *L*_*x*_ = 33.2 Å, *L*_*y*_ = 52.8 Å, and *L*_*z*_ = 26.8 Å. The MIL-53(Cr) framework was described by our previously derived flexible forcefield, including intra-molecular and inter-molecular potential terms^34^. CH_4_, CO_2_, N_2_ were treated with the standard microscopic models reported in the literature^36–38^. The MIL-53(Cr)/gas molecules interactions were treated by a Lennard–Jones (LJ- and Coulombic terms published elsewhere^34^ with the cross-term LJ parameters obtained using the mixing Lorentz–Berthelot rules). Ewald summation was used for calculating the electrostatic interactions, and the short-range interactions were truncated at 12 Å. The HOMC corresponds to a GCMC scheme combined with a MD move in *NσT* ensemble (where *N* is the number of molecules, *T* the temperature and *σ* the isotropic constraint) to allow the anisotropic changes of both shape and volume of the MOF framework^39^. The Nose–Hoover thermostat and barostat were used with a relaxation time of 0.5 ps. MD moves were conducted for 100 ps with a time step of 2 fs. The equations of motion were integrated using the velocity Verlet scheme. The MC procedure in the grand canonical ensemble corresponds to 5000 steps such that the ratios for each trial moves were defined as follows: 0.2004 for the translation, 0.2004 for the rotation, 0.599 for the insertion/deletion and 0.0002 for the MD move. A HOMC run corresponds to 100 steps corresponding to a sampled MD time of 1.0 ns and 500,000 MC steps. To validate our findings, several HOMC simulations with different MD and MC steps pairs have been tested in order to confirm that the simulations do not depend on the MC or MD steps number. In addition, we have conducted a very long time simulation (1 × 10^5^ HOMC steps i.e., ~100 ns and 500 × 10^6^ MC steps) to confirm the stability of the HOMC algorithm.

## Supplementary information

Supplementary Information

Supplementary Figure 1

Supplementary Figure 2

Supplementary Figure 3

Supplementary Figure 4

## Data Availability

The data that support the findings of this study are available on request from the corresponding author.
