# Author Correction: Tailoring the separation properties of flexible metal-organic frameworks using mechanical pressure

**DOI:** 10.1038/s41467-021-21523-7

**Published:** 2021-02-15

**Authors:** Nicolas Chanut, Aziz Ghoufi, Marie-Vanessa Coulet, Sandrine Bourrelly, Bogdan Kuchta, Guillaume Maurin, Philip L. Llewellyn

**Affiliations:** 1 Aix-Marseille University, CNRS Laboratoire MADIREL (UMR7246), Marseille, France; 2 Massachusetts Institute of Technology, MultiScale Materials Science for Energy & Environment <MSE 2>, MIT-CNRS-AMU joint laboratory/MIT Energy Initiative, Cambridge, 02139 MA USA; 3 Institut de Physique de Rennes, IPR UMR 6251 CNRS, Rennes, France; 4 Department of Chemistry, Wroclaw University of Science and Technology, Wroclaw, Poland; 5 Institut Charles Gerhardt Montpellier, UMR 5253 CNRS, UM, ENSCM, University of Montpellier, Montpellier, France

**Keywords:** Carbon capture and storage, Metal-organic frameworks

Correction to: *Nature Communications* 10.1038/s41467-020-15036-y, published online 5 March 2020.

The original version of this Article contained an error in the spelling of the author Bogdan Kuchta, which was incorrectly given as Bodgan Kuchta. This has now been corrected in both the PDF and HTML versions of the Article.

