# Supplementary material for: Tailoring the separation properties of flexible metal-organic frameworks using mechanical pressure
Source: Nat Commun. 2020 Mar 5;11:1216. doi: 10.1038/s41467-020-15036-y (PMC7058087; doi:10.1038/s41467-020-15036-y)
Supplement: Supplementary file 1 — Supplementary Information [file 41467_2020_15036_MOESM1_ESM.pdf]

Supporting Information

Tailoring the separation properties of flexible metal-organic frameworks using mechanical pressure

Chanut, et al.

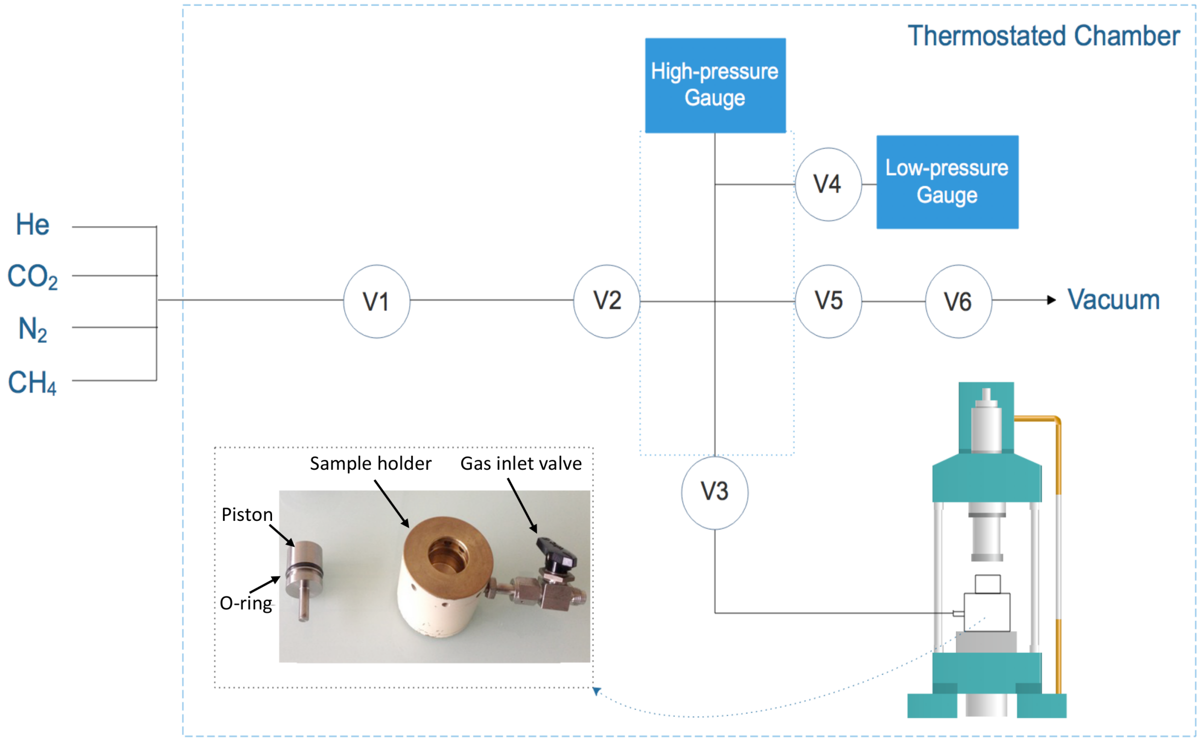

Supplementary Figure 1 | Experimental setup. Schematic representation of the adsorption manometry system used for the measurement of adsorption isotherms under mechanical constraint. A gas dose is introduced into the reference volume through valve V2 and allowed to stabilize before being brought into contact with the sample by opening valve V3. The pressure in the measurement manifold is monitored with both a low-pressure gauge (to 1bar) and a high-pressure gauge (from 1 to 40 bar) for optimal accuracy on the entire pressure range. **Photo,** Sample cell developed in order to maintain the gas pressure when applying an external mechanical pressure. The sample is placed in the sample holder (surface = 0.5 cm^2), a piston is used to apply the mechanical pressure on the sample, an O-ring situated on the piston allows maintaining the system leak tight to gas pressure (up to 20 bars) and a valve system allows vacuum and gas input. The entire experimental setup is situated in a thermostated chamber with the sample cell placed under the hydraulic press and connected to the gas dosing system.

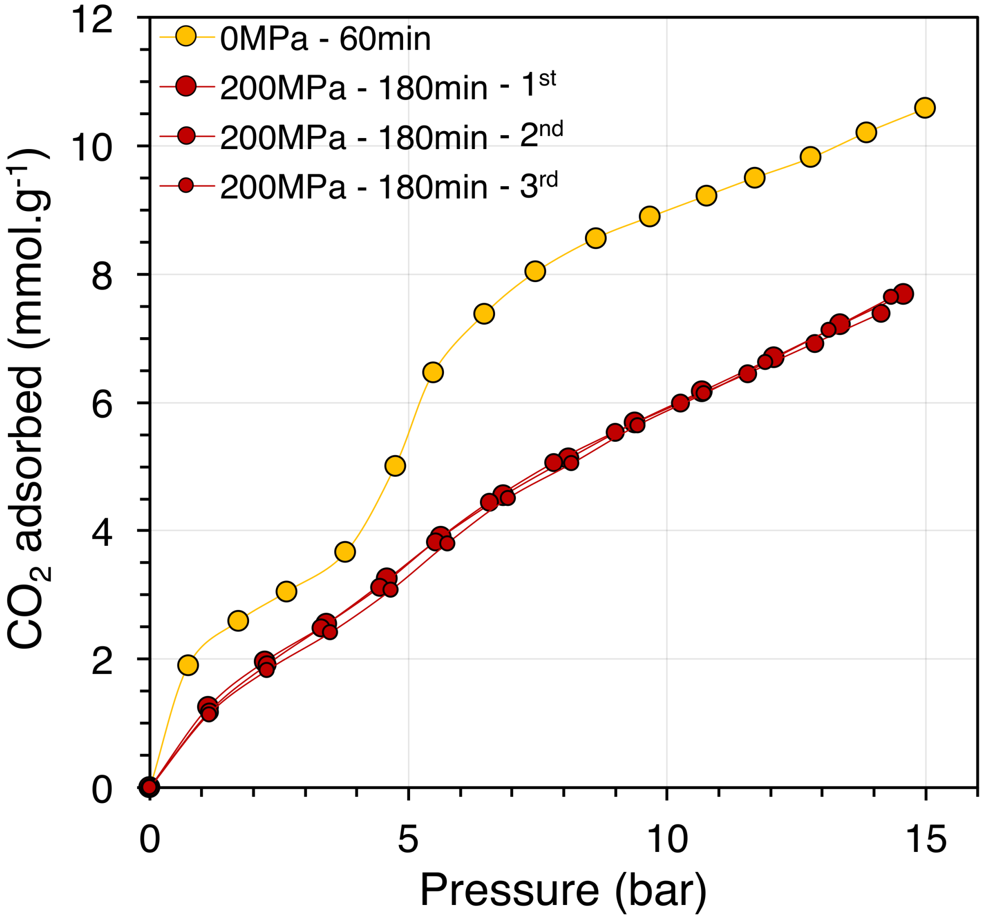

Supplementary Figure 2│Reproducibility of CO_2 adsorption isotherm measurements. Comparison of CO_2 adsorption isotherms at 303K under 200 MPa of mechanical constraints with release of the mechanical pressure between each measurement. CO_2 adsorption isotherm at 303K for the unconstrained material is given for comparison. Yellow and red filled circles represent applied mechanical pressure of 0 and 200 MPa with an equilibrium time is set at 60 and 180 minutes, respectively. Red filled circles of decreasing sizes are used to differentiate the first, second and third reproducibility measurement respectively.

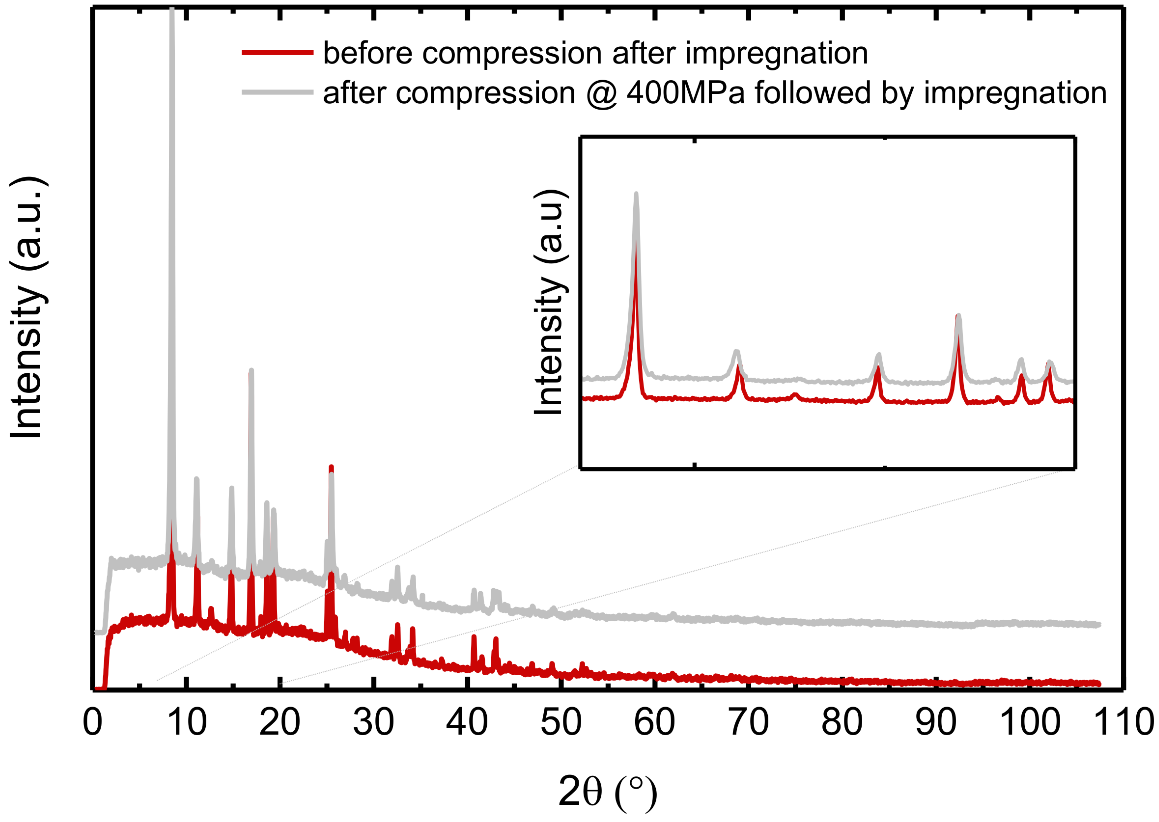

Supplementary Figure 3│ Stability of the MIL-53(Al) after compression/decompression cycles. X-Ray Diffraction patterns measured after impregnation in ethanol for the sample before compression and for a sample after 300 compression cycles up to 400MPa. Ethanol is used to fully open the structure (LP phase) and avoid any LP/NP phase coexistence. The comparison of the two diffractograms indicates that the sample does not show any significant structural degradation after these extensive studies.

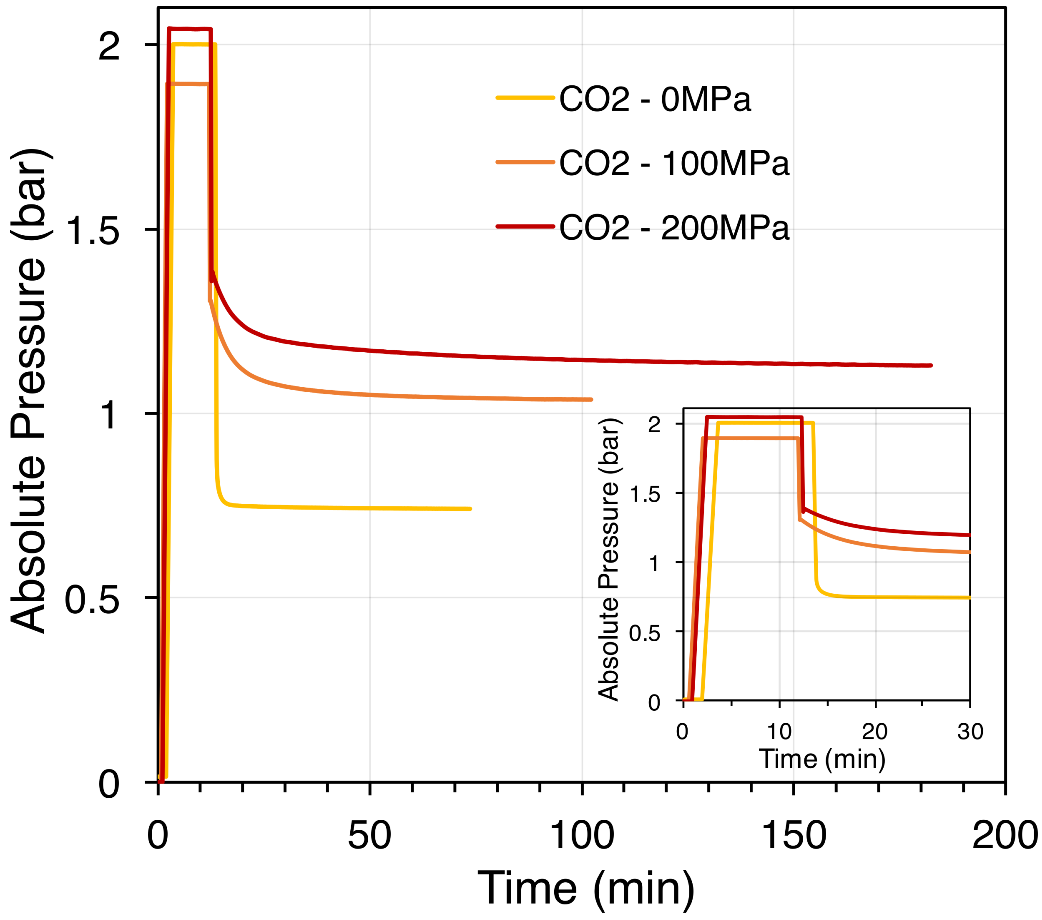

Supplementary Figure 4 | Adsorption kinetic of CO_2 under mechanical constraint. Evolution of pressure as a function of time for the first CO_2 dose of high-pressure adsorption isotherms under 0 MPa (yellow), 100 MPa (orange) and 200 MPa (red). The insert represents a zoom on the 30 first minutes of the CO_2 dosing process. We consider that the equilibrium has been reached when the change in pressure is less than 0.0005 bar over the last 10 minutes.
