# Supplementary figures and images for: Tailoring the separation properties of flexible metal-organic frameworks using mechanical pressure
Source: Nat Commun. 2020 Mar 5;11:1216. doi: 10.1038/s41467-020-15036-y (PMC7058087; doi:10.1038/s41467-020-15036-y)

# Thermostated Chamber

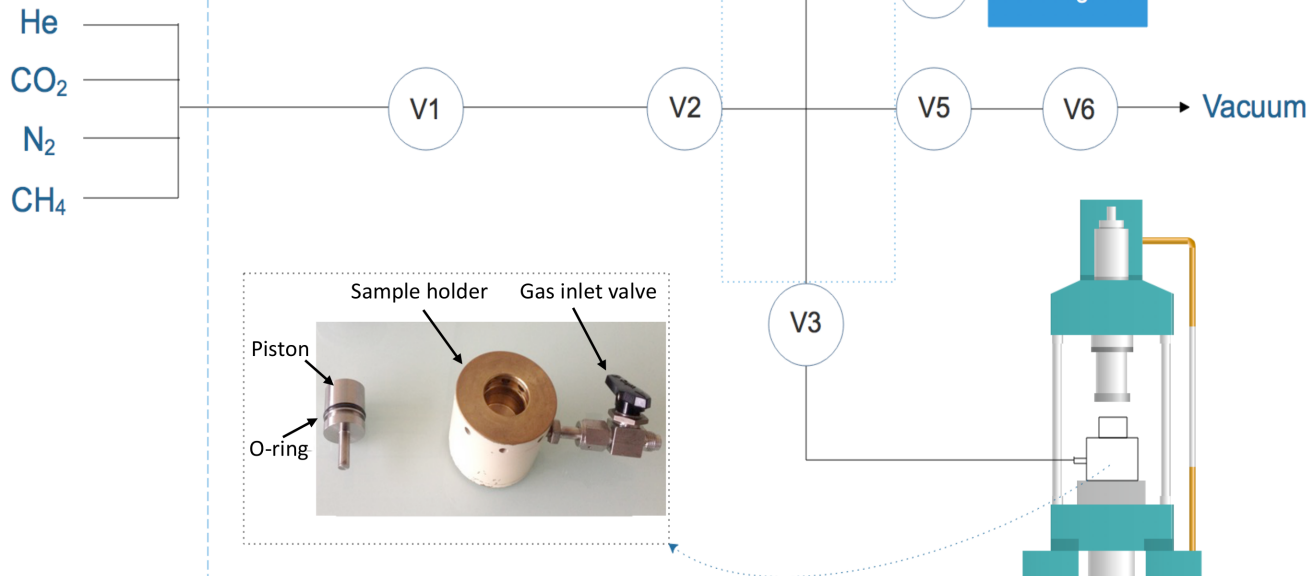

Supplement: Supplementary file 2 — Supplementary Figure 1 [file 41467_2020_15036_MOESM2_ESM.pdf]

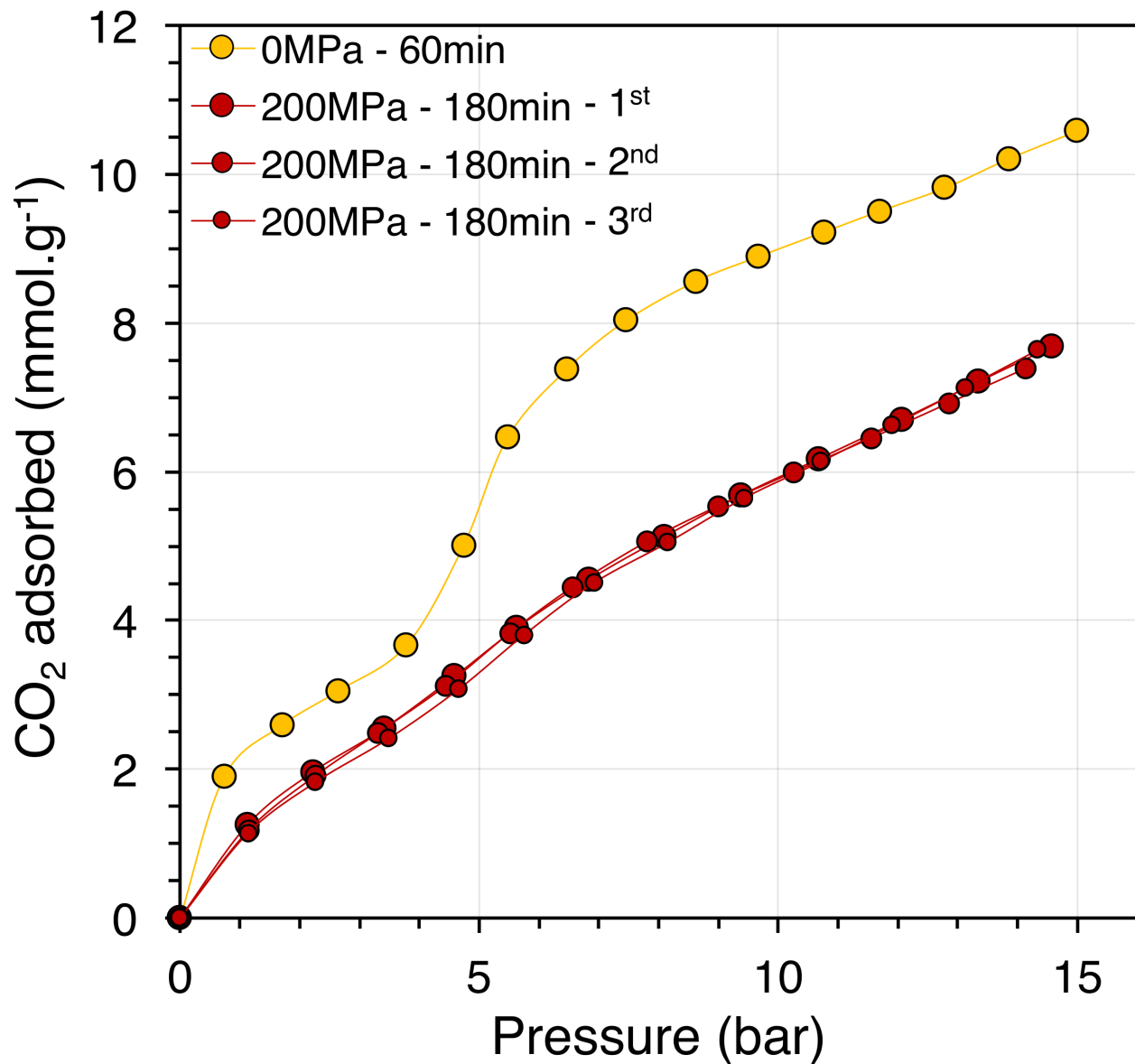

Supplement: Supplementary file 3 — Supplementary Figure 2 [file 41467_2020_15036_MOESM3_ESM.pdf]

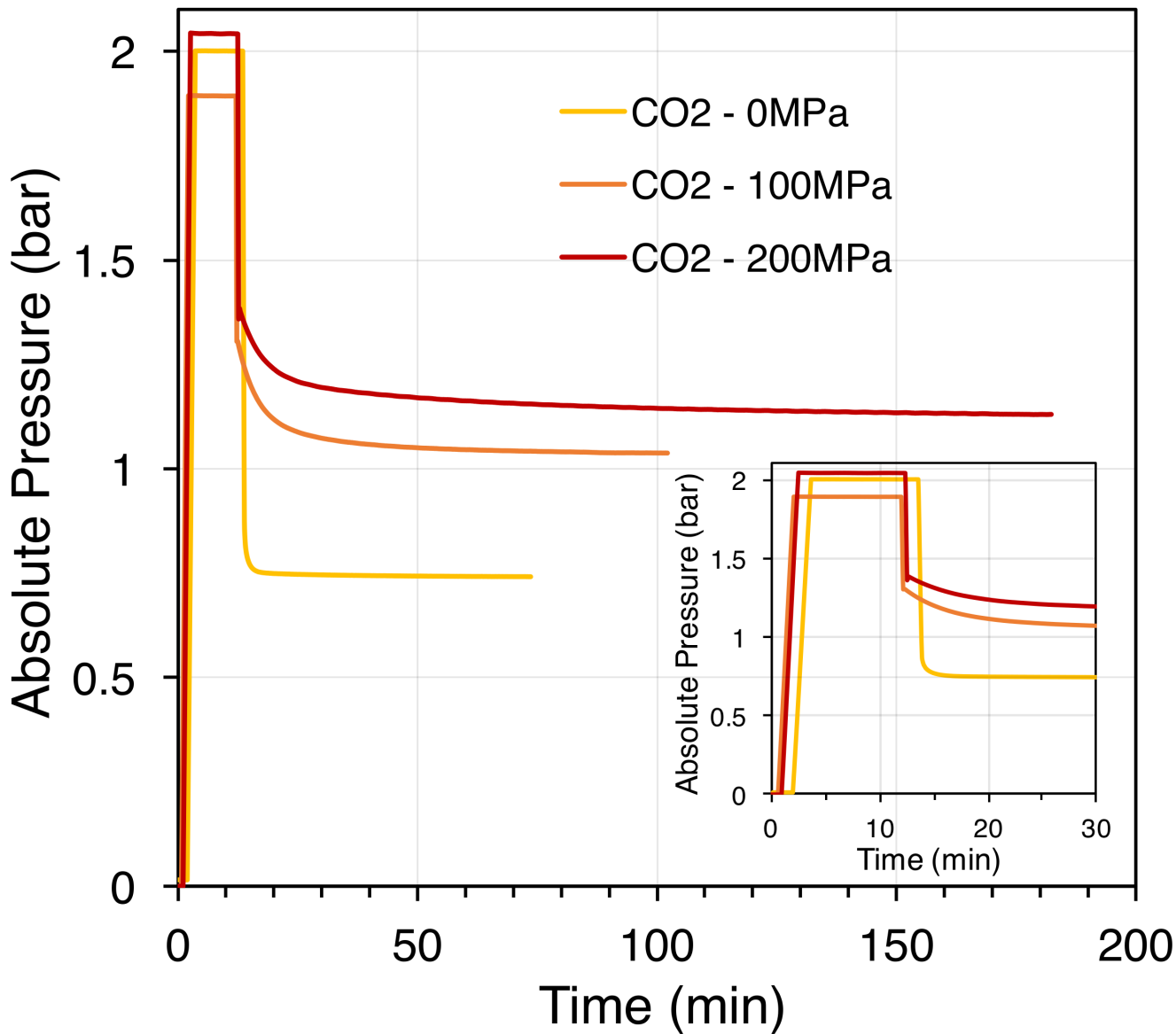

Supplement: Supplementary file 5 — Supplementary Figure 4 [file 41467_2020_15036_MOESM5_ESM.pdf]
