# Supplementary material for: Tailoring the separation properties of flexible metal-organic frameworks using mechanical pressure
Source: Nat Commun. 2020 Mar 5;11:1216. doi: 10.1038/s41467-020-15036-y (PMC7058087; doi:10.1038/s41467-020-15036-y)
Supplement: Supplementary file 4 — Supplementary Figure 3 [file 41467_2020_15036_MOESM4_ESM.pdf]

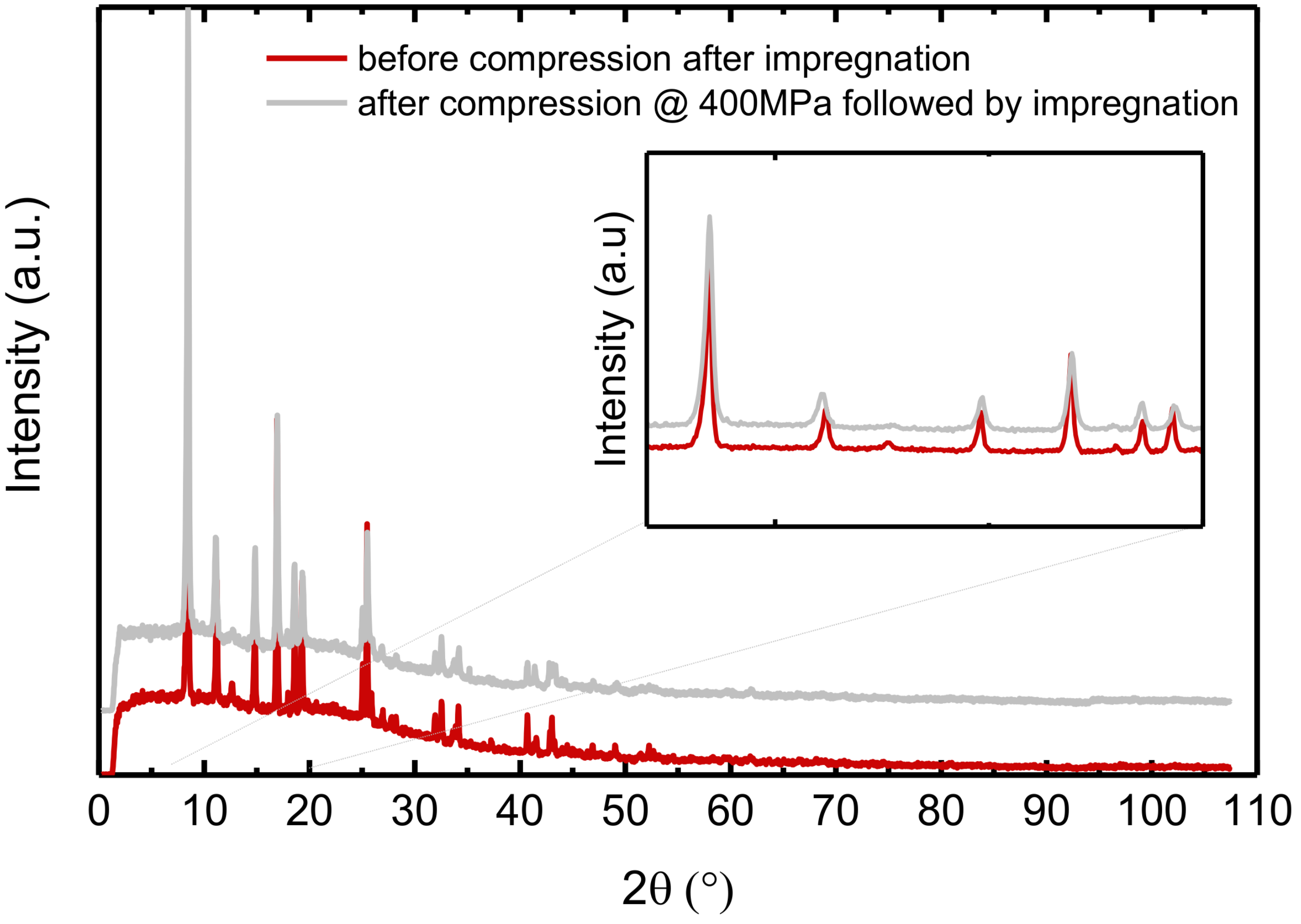

before compression after impregnation
after compression @ 400MPa followed by impregnation
Intensity (a.u.)
Intensity (a.u)
0
10
20
30
40
50
60
70
80
90
100
110
2θ (°)
